# Impact of Mutations in the *NCAPG* and *MSTN* Genes on Body Composition, Structural Properties of Skeletal Muscle, Its Fatty Acid Composition, and Meat Quality of Bulls from a Charolais × Holstein F_2_ Cross

**DOI:** 10.3390/ijms27020882

**Published:** 2026-01-15

**Authors:** Elke Albrecht, Praveen Krishna Chitneedi, Dirk Dannenberger, Christa Kühn, Steffen Maak

**Affiliations:** 1 Research Institute for Biology of Farm Animals (FBN), 18196 Dummerstorf, Germany; elke.albrecht@fbn-dummerstorf.de (E.A.); chitneedi@fbn-dummerstorf.de (P.K.C.); dannenberger@fbn-dummerstorf.de (D.D.); christa.kuehn@fli.de (C.K.); 2 Friedrich-Loeffler-Institute (FLI), 17493 Greifswald-Insel Riems, Germany; 3 Faculty of Agricultural and Environmental Sciences, University of Rostock, 18059 Rostock, Germany

**Keywords:** cattle, MSTN-Q204X, NCAPG-I442M, carcass quality, meat quality, muscle fiber, marbling, adipocyte, fatty acid composition

## Abstract

Cattle breeds are optimized either for milk or meat production and secrete consumed nutrients in the form of milk or accrete nutrients as skeletal muscle tissue, respectively. Surplus energy is usually stored in the form of fat in adipose tissues. To gain more insight into the physiological and genetic background of nutrient accretion as either protein or fat, an experimental F_2_ population was generated crossing Charolais (CH) bulls and German Holstein (GH) cows. Mutations in two genes with known, profound effects on growth were segregating in this population: the I442M mutation in the non-SMC condensin I complex, subunit G (*NCAPG*) gene, and the Q204X mutation in the myostatin (*MSTN*) gene. The major aim of this study was to close the gap between the described effects of the *NCAPG/LCORL* region and *MSTN* SNPs on carcass and meat quality traits, as well as on the structure and composition of the underlying tissues. Whole carcass data, meat quality traits, composition of major cuts and their dominating muscles, including muscle and fat cell structure, were analyzed as well as chemical and fatty acid composition. Mutant alleles of both loci were associated with higher weights, increased muscularity, and reduced fatness, e.g., each explaining about 15% of the observed variance. However, both loci apparently affect traits in a specific manner, influencing either dimensional traits or mass accretion.

## 1. Introduction

Most cattle breeds nowadays are selected for a single purpose, optimized either for milk or meat production. Thus, meat-type breeds, such as Charolais, preferentially accrete consumed nutrients as skeletal muscle tissue, whereas dairy breeds, such as Holstein-Friesian, secrete more nutrients in the form of milk. Dairy breeds usually store surplus energy, which is not needed for milk production, in the form of fat in adipose tissues [1]. The type of body mass accretion as either fat or protein is crucial for the efficiency of meat production. On the other hand, the quality of meat depends on the cellular structure and the amount and distribution of incorporated fat in the muscle tissue [2]. To gain more insight into the physiological and genetic background of nutrient accretion as either protein or fat, an experimental F_2_ population was created by crossing Charolais (CH) bulls and German Holstein (GH) cows, representing the accretion and secretion types, respectively, as described in detail by Kühn et al. [3]. The two breeds were chosen because of their differences in milk and meat yield, while being of similar size and maturing age and having significant economic importance [3]. Moreover, mutations in two genes with known, profound effects on growth were segregating in this population: the I442M mutation in the non-SMC condensin I complex, subunit G (*NCAPG*) gene, and the Q204X mutation in the growth differentiation factor 8 or myostatin (*GDF8/MSTN*) gene [4,5]. Both single-nucleotide polymorphisms (SNPs) were closely related to parameters of growth during different ontogenetic periods, adipose tissue deposition, body measures, and feed efficiency in this population [4,5,6,7,8]. Metabolomic analyses revealed that both mutations independently altered metabolic pathways linked to proportional and nonproportional growth patterns at puberty. The *NCAPG* I442M mutation was linked to arginine metabolism and proportional growth parameters, whereas the Q204X mutation of the *MSTN* gene was related to variations in free carnitine plasma levels, levels of several species of glycerophosphatidylcholines, and nonproportional growth [5]. Additionally, both mutations had significant effects on feed efficiency parameters. By combining the SNPs with metabolite data, Widmann et al. [8] hypothesized a functional relevance of sphingolipids for energy metabolism in growing cattle.

Mutations in the *MSTN* gene, which are often apparent in cattle breeds that were selected for high muscle content of the carcass, are important for the regulation of muscle fiber development and growth [9]. The Q204X mutation (c.615 C>T; rs110344317), frequently found in CH cattle, results in a premature stop codon in the N-terminal latency-associated peptide domain [9], and the mutant allele is therefore a disruptive variant. Animals carrying two copies of inactive alleles are designated as double-muscled cattle, because they have dramatically increased muscle mass, improved dressing percentage, and reduced carcass fatness [9,10]. However, important adverse effects on fitness (dystocia, stress susceptibility, fertility) have been reported in double-muscled cattle [11]. Heterozygous carriers in the CH population have better estimated breeding values for carcass weight and conformation [12]. While several studies have concordantly indicated positive effects of the Q204X mutant allele on meat production-related traits, inconsistent results regarding birthweight have been reported [13,14].

The I442M (c.1326T>G, rs109570900) mutation of the bovine *NCAPG* gene was identified as a promising candidate quantitative trait nucleotide (QTN) for carcass weight and fetal growth parameters in a quantitative trait locus (QTL) region on chromosome 6 in 2009 [4,6]. Subsequent studies in different cattle populations cast doubts on the QTN role of the *NCAPG* SNP and instead pointed to SNPs in or in close vicinity to the *LCORL* (ligand-dependent nuclear receptor corepressor-like) gene as putative causative variants [12,15]. Besides the above-mentioned investigations, the genome region spanning the *LCORL/NCAPG* genes has been associated with growth- and height-related parameters in different farm animal species [16,17,18,19]. Although recent studies have excluded this specific SNP as the causative variant for carcass and growth traits in cattle [20,21], we used the *NCAPG* I442M SNP for grouping our population because it was genotyped in all bulls and is in very strong linkage disequilibrium with putative causal variants in the *LORCL-NCAPG* region.

The major aim of this study was to characterize the effects of *MSTN* and *NCAPG* SNPs on carcass and meat quality traits, as well as parameters of structure and composition of the underlying tissues. Consequently, we analyzed the relationship between both mutations and muscle structure, fat cell parameters, and muscle composition in bulls of our CH × GH-based F_2_ population.

## 2. Results

### 2.1. Weights and Carcass Composition

**Figure 1:**
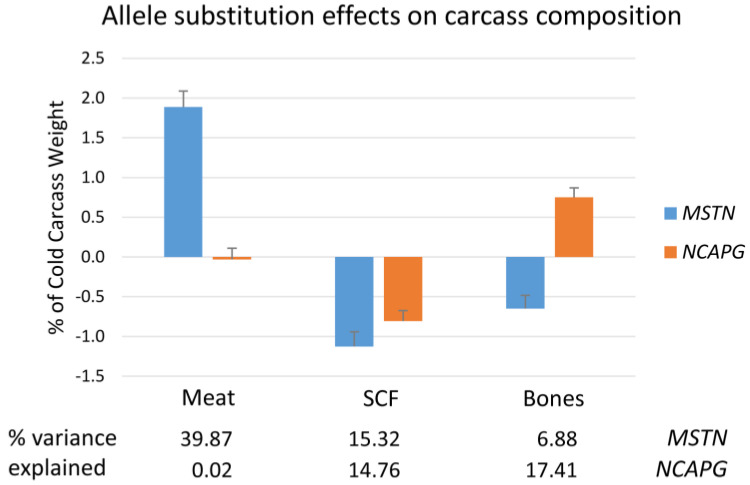
Allele substitution effects of *MSTN* Q204X and *NCAPG* I442M mutation on the content of meat, subcutaneous fat (SCF), and bones in the carcass of F_2_ bulls from a Charolais × Holstein cross population.

The *NCAPG* I442M mutation was present in all allele combinations, whereas only homozygous wild-type and heterozygous mutant animals were observed for the Q204X mutation at the *MSTN* locus. The allele frequencies were 0.51 for the *NCAPG* I442M mutation and 0.16 for the *MSTN* Q204X mutation in our population. Both mutations exerted strong effects on body size and composition (Figure 1 and Supplemental Table S1). Mutations at both loci were related to an increased birthweight, explaining 3.3% (*MSTN*) and 11.9% (*NCAPG*) of the phenotypic variance in our F_2_ population. In contrast, slaughter weight and carcass length were exclusively affected by the *NCAPG* mutation (10.7% and 10.6% of phenotypic variance, respectively), while more than 40% of the variance in dressing percentage was explained by the *MSTN* mutation. Moreover, carcass composition was influenced by both mutations, reducing fat deposition, in particular omental, perirenal, and subcutaneous, and increasing either the amounts of meat (*MSTN*) or bones and tendons (*MSTN* and *NCAPG*). In contrast, the *MSTN* Q204X mutation reduced the amount of bones in the carcass by 0.6% points. The *MSTN* Q204X mutation explained 39.9% and 31.1% of the variance in meat and protein content of the carcass, respectively. The amount of mesenteric and omental fat was mainly affected by the *NCAPG* mutation, explaining 4.4% and 11.2% of the variance, respectively. An increase in carcass protein and a decrease in carcass fat were observed for both mutations. Accordingly, daily weight gain of protein and fat was influenced by both mutant loci, but to a different extent.

### 2.2. Dimensions, Weights, and Chemical Composition of Carcass Parts

**Figure 2:**
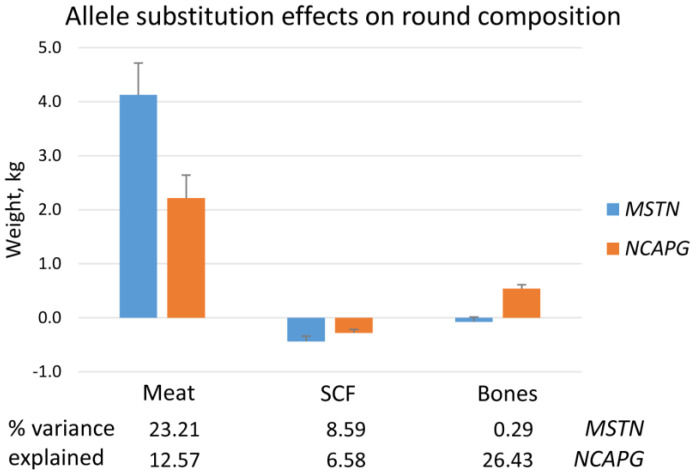
Allele substitution effects of *MSTN Q204X* and *NCAPG I442M* mutation on the composition of the round of F_2_ bulls of a Charolais × Holstein cross population. SCF—subcutaneous fat.

The results for whole carcass data were reflected in two major parts of the carcass—the back and the round (Figure 2 and Supplemental Table S2). Both mutations were related to increased weights of the carcass parts, more meat, increased protein accretion, water and ash content, and decreased fat content of the parts. The *NCAPG* I442M mutation had stronger effects on parameters defining dimensional traits, like the length of the round, than the *MSTN* locus. This is supported by the exclusive effect of the *NCAPG* mutation on the amount of bones in both carcass parts, explaining 11.2% and 26.4% of the variance in this trait in back and round, respectively. In contrast, the *MSTN* Q204X mutation exerted larger effects on mass accretion, in particular on the weight of meat in the back and round, explaining 10.2% and 23.2% of the variance, respectively. The distinct effects of both mutations on components of the carcass parts are exemplarily illustrated for the round in Figure 2. The missing effects of both mutations on the amount of tendons in the back may be due to the generally lower prevalence of connective tissue in the back compared with the round. Both mutations were associated with an altered composition of the carcass parts, shifting from fat to lean tissue.

### 2.3. Muscle Traits and Meat Quality

**Figure 3:**
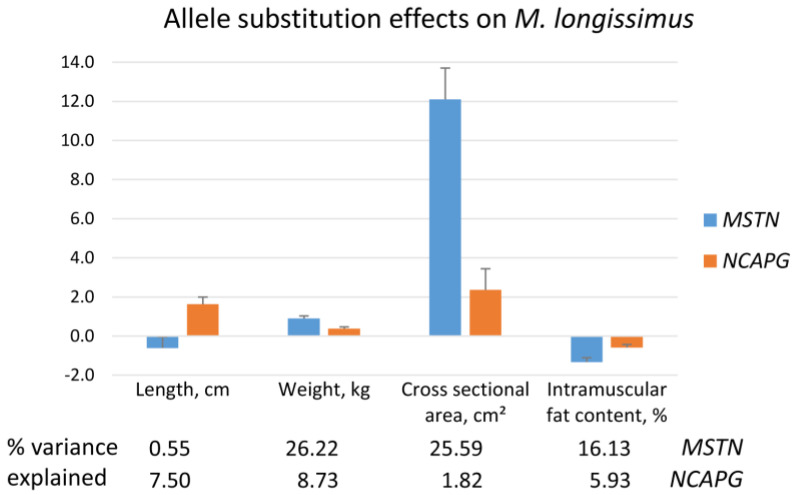
Allele substitution effects of *MSTN* Q204X and *NCAPG* I442M mutation on *M. longissimus* traits of F_2_ bulls of a Charolais × Holstein cross population.

Parameters of two major muscles contributing to the carcass parts back (*M. longissimus*) and round (*M. semitendinosus*) are provided in Supplemental Table S3. Muscle weight and content of intramuscular fat (IMF) of *M. longissimus* were influenced by both mutations, leading to heavier muscles with reduced IMF content in bulls with at least one mutant allele at *MSTN* or *NCAPG*, respectively. While the *NCAPG* mutant allele increased the length of *M. longissimus* by 1.6 cm, the *MSTN* Q204X mutation was stronger related to a larger cross-sectional area of the muscle, explaining 25.6% of the variance in this trait (Figure 3). Both mutations decreased the IMF content also in *M. semitendinosus*, but only the *MSTN* mutation affected meat quality traits, such as lightness and shear force after 14 days, leading to a brighter color and reduced tenderness. In contrast, the moderate increase in shear force after 14 days in *M. longissimus* was related to the mutant alleles of both genes. The pH value of *M. longissimus* was marginally reduced in bulls with the mutant *MSTN* allele; thus, no tendency toward DFD (dark, firm, dry) meat was observed.

### 2.4. Cellular Structure of the M. longissimus

**Figure 4:**
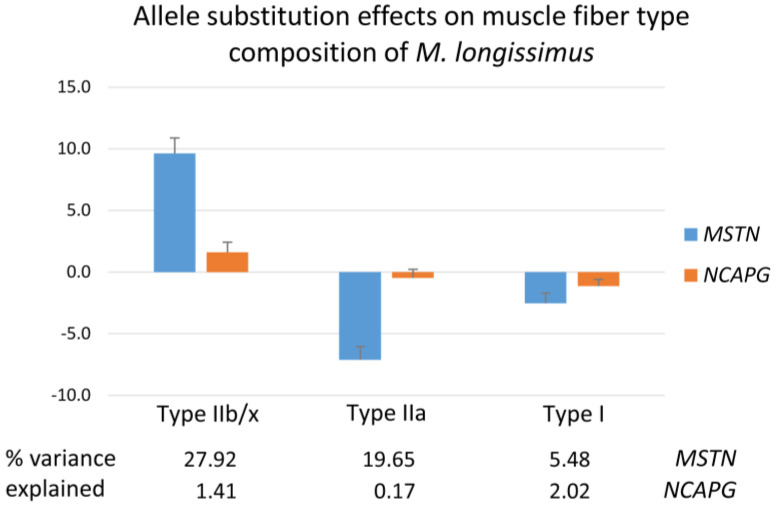
Allele substitution effects of *MSTN* Q204X and *NCAPG* I442M mutations on muscle fiber type composition in *M. longissimus* of F_2_ bulls of a Charolais × Holstein cross population.

To elucidate underlying changes at cellular level, *M. longissimus* muscle fiber size (muscle fiber cross-sectional area—CSA) and composition (area percentage of the fiber types), as well as intramuscular fat deposition, were analyzed. There were partly different effects of both mutations on muscle fiber CSA and composition (Figure 4 and Supplemental Table S4). The muscle fiber CSA was reduced in *M. longissimus* of bulls with the *NCAPG* I442M mutation, leading to an increased fiber number per cm^2^. There was no significant effect of the *MSTN* genotype on the overall muscle fiber CSA due to opposite effects on the size of glycolytic and oxidative muscle fibers. Type IIb/x fibers were larger, whereas type IIa fibers were smaller in carriers of the *MSTN* Q204X mutation. The apparent total fiber number, calculated as muscle cross-sectional area × muscle fiber number/cm^2^, was significantly affected by both mutant alleles, owing to the fact that the *MSTN* Q204X mutation was positively related to the cross-sectional area of the muscle. There was a significant negative effect of the mutant alleles of both genes on the CSA of type IIa muscle fibers, but the fiber type composition of the muscle, described by the area percentage of all three fiber types, was mainly influenced by the *MSTN* mutation, as illustrated in Figure 4. Carriers of the mutant allele had a higher proportion of type IIb/x fibers and a lower percentage of the other fiber types. About 27.9% of the variance in the area percentage of IIb/x fibers and 19.7% of type IIa fibers was explained by the *MSTN* mutation (Figure 4). Both mutations reduced the size of intramuscular fat cells as well as the marbling (visible IMF) area percentage, consistent with the observed lower extractable IMF content of the muscle in bulls with at least one mutant allele (Supplemental Tables S3 and S4). The maximum size of marbling flecks was reduced, and the distance between them, as an indicator of marbling fleck distribution, was enlarged by the *MSTN* mutation, while both mutations reduced the number of marbling flecks.

### 2.5. Fatty Acid Composition of the M. longissimus

**Figure 5:**
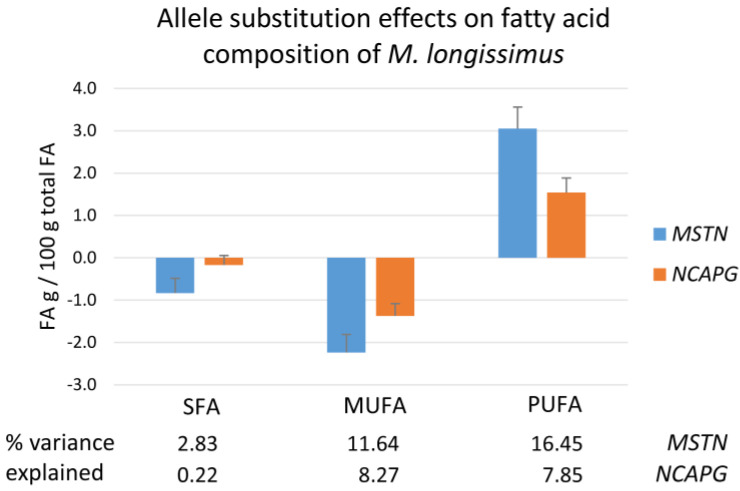
Allele substitution effects of *MSTN* Q204X and *NCAPG* I442M mutations on fatty acid composition in *M. longissimus* of F_2_ bulls of a Charolais × Holstein cross population. SFA—saturated fatty acids, MUFA—monounsaturated fatty acids, and PUFA—polyunsaturated fatty acids.

Besides the described decreasing effects of both mutant alleles on the fat content of *M. longissimus*, there were significant effects of both mutations on the fatty acid composition of the muscle (Figure 5 and Supplemental Table S1). The proportion of saturated fatty acids (SFAs) was decreased by the *MSTN* mutation only, explaining 2.8% of its variance. The content of polyunsaturated fatty acids (PUFAs) was higher, and that of monounsaturated fatty acids (MUFAs) was lower, in carriers of mutations at both loci, but with stronger effects of the *MSTN* Q204X mutation. The increase in PUFA concerned both n-3 and n-6 PUFA. However, the effect on the n-6/n-3 PUFA ratio was significant for the *MSTN* mutation only, leading to a higher ratio in *M. longissimus* of heterozygous compared with wild-type bulls. A detailed overview of the effects of both mutations on the fatty acid composition of *M. longissimus* is given in Supplemental Table S5.

## 3. Discussion

We provide here a comprehensive analysis of the effects of two mutations in the *MSTN* (Q204X) and the *NCAPG* (I442M) gene at different phenotypic levels, from carcass to cellular level, in a Charolais- and German Holstein-based F_2_ population. Besides whole carcass data and meat quality traits, major cuts and their dominating muscles were analyzed for structural and compositional traits, including muscle and fat cell structure, as well as chemical and fatty acid composition. We have shown that the reported relationships between SNPs in the *MSTN* and *NCAPG* loci and growth and carcass traits in cattle are associated with distinct structural differences in the underlying skeletal muscle and adipose tissues.

Our data on birthweight and carcass traits in groups with different alleles at both loci largely confirm the well-described associations for *MSTN* [14,22,23] as well as for *NCAPG* [5,24,25]. Mutant alleles of both loci are associated with higher weights, increased muscularity, and reduced fatness. It was also confirmed that both loci apparently affect the traits in a specific manner. The *NCAPG* I442M mutation was associated with higher slaughter weights and longer carcasses, reflected by increased bone and tendon content in the whole carcass. Thus, it mainly influences dimensional traits in concordance with a former study in this population [5]. In contrast, the *MSTN* Q204X mutation is related to higher meat content and dressing percentage, thus influencing mass accretion, as also described by Allais et al. [13] for a French Charolais population. The features of the whole carcass were well reflected in the weights and dimensions of major parts of the carcass—the back and round—and their dominating muscles (*M. longissimus*; *M. semitendinosus*).

The increased muscularity in mutant animals was not generally accompanied by impaired meat quality traits (pH, shear force after 24 h, water-binding capacity). This was reflected by only few significant negative effects of both mutant alleles, e.g., on the tenderness of the *M. longissimus* after 14 days and the brightness of the M. semitendinosus. Allais et al. [13] observed similar effects for the *MSTN* Q204X allele in different beef breeds. In contrast, both mutant alleles influenced the chemical composition of both muscles in a similar manner. The increased growth of the muscles was facilitated by greater amounts of water and protein and a significant decrease in fat. The decreased overall fat content of the back was accompanied by diminished marbling of the *M. longissimus*. We observed very large effects of the mutations investigated in *MSTN* and/or *NCAPG*, explaining up to 40% of the phenotypic variance in some traits.

Effects of inactivating mutations of *MSTN* on muscle fiber development and properties have been studied in model and farm animals in vitro and in vivo [13,26,27,28,29]. However, most studies compared animal homozygous for inactivating mutations of *MSTN* with wild-type controls, in contrast to this study, which focused on heterozygous carriers of the *MSTN* Q204X mutation. Nevertheless, the observed increased proportion of type IIb/x fibers and the decreased proportion of type I and type IIa fibers in heterozygous bulls conform to results in *MSTN* k.o. mice [27]. Hayashi et al. [28] reported a downregulation of the myosin 2x heavy chain by MSTN in normal bovine myoblasts, in contrast to myoblasts derived from homozygous mutant animals. This could explain the observed shift in myofiber types in our study, which resulted in a significantly brighter muscle color in *M. semitendinosus*. The mean cross-sectional area of all fiber types was not clearly affected by *MSTN* in our F_2_ population because of opposite effects on individual fiber types (Supplemental Table S4). Type IIb/x fibers were larger and type IIa fibers were smaller in *M. longissimus* of heterozygous bulls, further indicating a change to a more glycolytic muscle metabolism. In contrast, Allais et al. [13] described a significant decrease in total muscle fiber size in a large cohort of purebred Charolais bulls heterozygous for *MSTN* Q204X. Nevertheless, they reported the average cross-sectional area of all fiber types as 2920 µm^2^, similar to the values measured in this study.

Together with the increased muscle cross-sectional area, this indicated a more pronounced muscle fiber hyperplasia, which was confirmed by an increased apparent total muscle fiber number in bulls with the mutant *MSTN* allele compared to wild-type ones in our investigation. Muscle fiber hypertrophy of type IIb/x muscle fibers additionally contributes to the enlarged muscle cross-sectional area. The unchanged number of nuclei per muscle fiber in both genotypes supports the observations of Amthor et al. [30] and Lee et al. [29] that the muscle growth driven by myostatin inactivation does not involve depletion of the stem/precursor cell reservoir.

The diameter of the intramuscular adipocytes in the *M. longissimus* was smaller in *MSTN* mutant bulls, and the number of marbling flecks was reduced; thus, the distance between the marbling flecks became greater. This is in accordance with the lower intramuscular fat content in heterozygotes observed by Allais et al. [13] and in this study and has probably contributed to the observed increase in shear force after 14 days. The decreased intramuscular fat content was associated with smaller marbling flecks that usually contain smaller adipocytes, as observed in the study of Yang et al. [31]. Fewer, smaller, and unevenly distributed marbling flecks cannot fulfill their positive influence on taste, juiciness, and tenderness of meat; thus, the overall meat quality may be impaired [2].

In contrast to *MSTN* effects, data on relationships between mutations at the *LCORL/NCAPG* locus and cellular parameters of the muscle have not been reported so far. There was a significant effect of the *NCAPG* I442M mutation on muscle fiber size, in contrast to muscle fiber type composition. The decreased size and increased apparent total muscle fiber number indicated an extended muscle fiber hyperplasia in *M. longissimus*. The mutant allele was related to a significant decrease in the mean cross-sectional area across all fiber types, and in particular of type I and type IIa fibers. Again, in contrast to the *MSTN* effects, there was no significant change in fiber type composition and no change in type IIb/x diameter related to the *NCAPG* mutation. The effect on fat cell size was in the same direction as observed for the *MSTN* mutation. Although both mutations were related to similar effects at the carcass level—higher muscularity and lower fat content—apparently different mechanisms of muscle growth were targeted, while the effects on fat cells were similar.

Considering the standardized feeding of the bulls, the significant effects of both loci on some classes of fatty acids in the muscle indicate a direct impact on lipid metabolism. However, the observed increase in the n-6/n-3 PUFA ratio in animals with the *MSTN* mutation is not desired from the perspective of improving meat quality for human nutrition, because a targeted higher proportion of n-3 PUFA would result in a lower n-6/n-3 PUFA ratio [32]. Carriers of a mutant *MSTN* Q204X allele had an increased content of stearic acid (C18:0) with a concomitantly decreased content of the oleic acid isomer C18:1c9. A recent study identified a signaling cascade, *MSTN-MEF2C* (myogenic transcription factor 2C)-*miR222-SCD5* (stearoyl-CoA desaturase 5), regulating fatty acid desaturation and fat deposition in porcine adipocytes [33]. As *SCD5* is also expressed in bovine species, this mechanism is worth investigating further. This could explain a direct effect of MSTN inactivation on both muscle and adipose tissue. However, the reported involvement of palmitic acid (C16:0) and palmitoleic acid (C16:1) in this regulation cascade is not supported by our data, because the mutation was associated with decreased values for both fatty acids. Former results obtained from partial material of this F_2_ population linked the *MSTN* Q204X allele to circulating levels of diacylglycerophosphatidylcholines and thus indicated effects of the mutation on parameters of lipid metabolism [5]. Moreover, there were significant, similarly directed effects of the *NCAPG* mutation on the contents of C16:1 and C18:1c9, which cannot be functionally explained at present. In contrast to the results on muscle, the effects of both mutations on parameters of the lipid metabolism still await further elucidation.

While direct functional effects of *MSTN* on muscle growth have been well-established for decades (reviewed, e.g., by Lee [34,35] and Baig et al. [36]), recent studies have provided evidence for a direct involvement of *MSTN* in the regulation of lipid metabolism [33]. Thus, our results are largely in line with the current state of knowledge regarding *MSTN*’s role as master regulator of muscle and adipose tissue growth. The SNP investigated in this study is clearly designated as an inactivating mutation [9]. Consequently, the amount of functional myostatin is reduced in our heterozygotes throughout life, with the consequences discussed above. In contrast, the functional role of the *NCAPG* I442M mutation is still unclear. Recent studies demonstrated that *NCAPG* seems to be directly involved in myogenesis. A study in bovine fetal myoblasts revealed a crucial role of *NCAPG* in myogenesis, because knock-down of the gene impaired myogenesis by increasing apoptosis [37]. It was further shown that the *NCAPG* promoter effectively binds the myogenic transcription factors MYOD1 (myogenic differentiation 1) and the cAMP response element-binding protein 1 (CREB1) in bovine satellite cells [38]. It must be noted that both studies report a role of intact *NCAPG* in myogenesis, which contrasts with our observation that gradual inactivation of the gene (one or two mutant alleles) was related to increased muscularity and growth. However, muscle fiber size was decreased in animals carrying the mutant alleles. Thus, the actual mechanisms in cattle need further clarification. The *NCAPG* I442M mutation was originally considered as possible causative variant for several growth and carcass traits in different cattle populations [4,5,6,39] and was linked to variations in metabolic profiles [5]. Meanwhile, it is accepted that this SNP is in strong linkage disequilibrium with a “true” causative, yet to be identified variant in the *LCORL/NCAPG* locus [20,21,40]. A recent meta-analysis including our population identified six intronic SNPs, a missense variant, and a frameshift variant (rs 384548488) in the *LCORL* gene as lead SNPs for the QTL in this region, while no lead SNP was located in the *NCAPG* gene [20]. Majeres et al. [21] tried to narrow down the region of the causative SNP but failed because of the strong linkage disequilibrium in the *LCORL/NCAPG* genomic region. A different approach was used by Bai et al. [41], leading to the identification of the above-mentioned *LCORL* frameshift mutation (PRC2-associated LCORL isoform 2; *PALI2*) as the driver of the selective sweep in this region in cattle. The consequence of this SNP is a loss of the PIP (PALI interaction with PRC2) domain in this *LCORL* transcript. They further demonstrated that this mutation was subject to convergent artificial selection for large body size and fast growth in many domestic animal species [41]. Despite these results, the *NCAPG* I442M SNP deserves further consideration, as this variant is predicted to be deleterious [21] and the physiological consequences of the variant are not fully understood.

## 4. Materials and Methods

### 4.1. Animals

The 241 bulls used in this study were part of an F_2_ generation based on the founder breeds Charolais (CH) and German Holstein (GH). This population was established at the Research Institute for Farm Animal Biology (FBN, Dummerstorf, Germany). The experimental setup was described by Kühn et al. [3] and Pfuhl et al. [42]. Bulls were fattened under standardized conditions and were slaughtered at 18 months of age in the research institute’s experimental abattoir following a standardized protocol and ensuring uniform pre-slaughter handling. All carcasses were dissected and sampled for comprehensive phenotypic, physiological, metabolic, and genomic characterization. All animals were cared for and killed according to German rules and regulations for animal care. The Animal Protection Board of the FBN and the Animal Care Committee of the State Mecklenburg-Western Pomerania, Germany (State Office for Agriculture, Food Safety and Fishery; LALLF M-V/ Rostock, Germany, TSD/7221.3-2.1-010/03) approved the experiment.

### 4.2. Genotyping of the MSTN Q204X and NCAPG I442M Variants

All F_2_ individuals were genotyped for the *MSTN* c.160C>*T* (*MSTN* Q204X) genetic variant from blood DNA via a Tetra-ARMS assay as described by Weikard et al. [5]. The Tetra-ARMS assay was applied with first embracing primers (5′-3′): forward primer: AGACTCATCAAACCCATGAAAG (0.2 µM) and embracing reverse primer: TGAGTACAGGGCTACCACTGG (0.2 µM), followed by a specific primer pair: specific inner forward primer: ACTCAGGCACTGGTATTTGGT (0.2 µM) and specific inner reverse primer: ACTGTCTTCACATCAATACTCTG (1 µM). The annealing temperature for both PCRs was set at 60 °C.

The *NCAPG* c.1326T>G SNP (*NCAPG* I442M) was genotyped by PCR–RFLP as described by Eberlein et al. [4], using the restriction enzyme TasI (ThermoFisher Scientific, Waltham, MA, USA) and the amplification primers 5′-ATTTAGGAAACGACTACTGG-3′ (forward) and 5′-ATTTGTATTCTCTTATTATCATC-3′ (reverse).

### 4.3. Carcass Characteristics

All carcasses were weighed and dissected following the protocol described by Pfuhl et al. [42] for purebred bulls. Various cuts of the carcass were dissected in meat, subcutaneous fat, bones, and tendons. Besides the whole carcass and adipose tissues, the current study focused on the round of beef and the back, the parts that contain the *M. semitendinosus* and *M. longissimus*, respectively, for further analyses. Fat and protein content of all cuts was determined individually after each respective tissue was ground, and a sample was analyzed by near-infrared spectroscopy using an Infratec 1255 Food & Feed Analyzer (Foss Analytical A/S, Hillerød, Denmark).

### 4.4. Meat Quality

Meat quality was determined for *M. longissimus* and *M. semitendinosu* as described in detail by Pfuhl et al. [42], using standard procedures for color, pH at 24 h (*M. longissimus*), shear force at 24 h and 14 d after slaughter, and forced water loss. The intramuscular fat content was measured in triplicate via the Soxhlet extraction method using petroleum ether as solvent and determined gravimetrically after evaporating the extracting solvent [43].

### 4.5. Muscle Structure

We used Cell^ image analysis software (ver. 3.4, 2011, OSIS, Münster, Germany) to determine muscle fiber size and composition, as well as marbling traits, in *M. longissimus* of 224 F_2_ crossbred bulls. For muscle fiber traits, data of fiber size (from H/E-stained section), fiber type (from ATPase-stained serial section), and the number of nuclei lying within a muscle fiber (from H/E-stained section) were combined as described by Albrecht et al. [44]. A minimum of 300 muscle fibers per animal was measured using an image analysis system equipped with a Jenaval microscope (Carl Zeiss, Jena, Germany), an Altra20 CCD camera (OSIS, Münster, Germany), and the muscle fiber measurement module developed by MAS (Freiburg, Germany) in the Cell^ software (OSIS). The apparent total muscle fiber number was calculated from the fiber number per cm^2^ and the muscle cross-sectional area. Marbling traits were determined in *M. longissimus* slices, which were fixed and stained with Oil Red-O and subsequently analyzed with Cell^ software (OSIS), as described by Albrecht et al. [44].

For measurement of intramuscular fat cell size in *M. longissimus* of F_2_ bulls, large marbling flecks were cut from muscle slices, frozen in liquid nitrogen, and used to prepare cryosections. These sections contained many fat cells that were measured with the interactive measurement module of the Cell^ software (OSIS). Fat cell size was determined as the average of about 300 adipocytes for each animal.

### 4.6. Fatty Acid Composition of M. longissimus

Fatty acid composition was determined in muscle samples of all F_2_ bulls as described by Kalbe et al. [32]. Briefly, after homogenization of frozen muscle samples and the addition of C19:0 as an internal standard, total muscle lipids were extracted in duplicate using chloroform/methanol (2:1, *v*/*v*) and an Ultra Turrax T25 (IKA, Staufen, Germany) at 3 × 15 s, 15,777 g, and room temperature. All solvents contained 0.005% (*w*/*v*) of t-butylhydroxytoluene to prevent oxidation of PUFA. To complete the extraction, the solutions were stored overnight in a refrigerator (4 °C). An aliquot of total lipids (25 mg) from each sample was used for methyl ester preparation. Transmethylation of fatty acids in the lipids was carried out using 0.5 M sodium methoxide in methanol, followed by 14% boron trifluoride in methanol as reagents. The fatty acid methyl esters (FAMEs) were extracted twice with 2 mL of n-hexane. Fatty acid analysis of the lipids was performed using capillary GC with a CP-Sil 88 CB column (100 m × 0.25 mm, Agilent, Santa Clara, CA, USA) installed in a PerkinElmer gas chromatograph CLARUS 680 with a flame ionization detector and split injection (PerkinElmer Instruments, Shelton, CT, USA). C19:0 was the internal standard for the quantification of fatty acids. The reference standard mixture “Sigma FAME” (Sigma-Aldrich, Deisenhofen, Germany), the methyl esters of C18:1cis-11, C22:5n-3, and C18:2cis-9,trans-11 (Matreya, State College, PA, USA), C22:4n-6 (Sigma-Aldrich, Deisenhofen, Germany), and C18:4n-3 (Larodan, Limhamn, Sweden) were used for the calibration procedure. Five-point calibration of single fatty acids ranged between 16 and 415 mg/mL and were assessed after GC analysis of five samples. Fatty acid proportions are given as g/100 g of total FA.

### 4.7. Statistical Analyses

The statistical analyses aimed to establish a correlation between carcass characteristic, meat quality, muscle structure, and fatty acid composition traits of *M. longissimus,* on the one hand, and genotypes at the *NCAPG* and *MSTN* loci, on the other. Normality of all data was performed using the Shapiro–Wilk test using the UNIVARIATE procedure in SAS statistical software (Version 9.4, SAS Inst., Cary, NC, USA). The association study was carried out with the genotyped loci of 241 animals and by taking advantage of the genomic relationship matrix (GRM), which was previously established using imputed high-density (HD) genotype data for the corresponding animals [20]. The statistical model y = 1μ + xb + g + e, implemented in GCTA [45], was based on a standard mixed linear model-based association (MLM) and was used to determine associations between the *NCAPG* genotype and different phenotypes. The vector of the tested trait is denoted by y, the population mean is represented by μ, the additive fixed effect of the tested variant by b, and the vector of allele for the tested variant (variable coded as 0, 1, or 2) by x. G represents the vector of random polygenic effects captured by g ∼ N (0, Gσ2g), where G is the genomic relationship matrix based on HD autosomal SNPs, and e represents the vector of residual variance captured by e ∼ N (0, Dσ2e), where D represents the identity matrix. The *MSTN* mutation segregated only in one family. By accounting for the entire genomic relationship matrix, we already included family effects. Thus, instead of MLM, a GCTA MLM leaving-one-chromosome-out (LOCO) analysis was implemented for this trait. The model y = 1μ + xb + g- + e is similar to the MLM model, except that g- represents the accumulated effect of all SNPs except those on the chromosome where the candidate SNP is located. The var(g-) was re-estimated each time a chromosome was excluded from GRM calculation. Furthermore, in order to account for the confounding effect of birth year across samples, birth year was included as a covariate in both MLM and MLM-LOCO analyses using GCTA’s -cov parameter and -mlma-no-preadj-covar option. The purpose of this option is to facilitate simultaneous fitting of covariates and SNP for the association test. This approach is intended to prevent the loss of power that can occur when phenotypes are pre-adjusted for covariates.

## 5. Conclusions

Mutant alleles of *MSTN* and *NCAPG* both are associated with higher weights, increased muscularity, and reduced fatness. However, both loci apparently affect these traits in a specific manner. Whereas the *NCAPG* mutation was associated with increased bone and tendon content in the whole carcass and mainly influenced dimensional traits, the *MSTN* mutation was related to higher meat content and dressing percentage, thus influencing mass accretion. At the cellular level, only the *MSTN* mutation influenced muscle fiber type composition. Furthermore, the significant effects of both loci on some classes of fatty acids in the muscle indicate a direct impact on lipid metabolism. Our results indicate that SNPs in the *MSTN* and *NCAPG* loci cause distinct structural differences in skeletal muscle and adipose tissues that lead to altered growth and carcass traits in cattle.

## Data Availability

The original contributions presented in this study are included in the article/Supplementary Material. Further inquiries can be directed to the corresponding author.

## Supplementary Materials

The following supporting information can be downloaded at: https://www.mdpi.com/article/10.3390/ijms27020882/s1.

## Abbreviations

The following abbreviations are used in this manuscript:

NCAPG	Non-SMC condensin I complex, subunit G
MSTN	Myostatin
FBN	Research Institute for Biology of Farm Animals
FLI	Friedrich-Loeffler-Institute
CH	Charolais
GH	German Holstein
GDF8	Growth differentiation factor 8
LCORL	Ligand-dependent nuclear receptor corepressor-like
SNP	Single-nucleotide polymorphism
QTN	Quantitative trait nucleotide
QTL	Quantitative trait locus
HCW	Hot carcass weight
CCW	Cold carcass weight
SD	Standard deviation
IQR	Interquartile range
SE	Standard error
SCF	Subcutaneous fat
*M. semitendinosus*	*Musculus semitendinosus*
*M. longissimus*	*Musculus longissimus dorsi*
IMF	Intramuscular fat
CSA	Cross-sectional area
SFA	Saturated fatty acids
MUFA	Monounsaturated fatty acids
PUFA	Polyunsaturated fatty acids
MEF2C	Myogenic transcription factor 2C
SCD5	Stearoyl-CoA desaturase 5
MYOD1	Myogenic differentiation 1
CREB1	cAMP response element-binding protein 1
PALI2	PRC2-associated LCORL isoform 2
PIP	PALI interaction with PRC2
FAME	Fatty acid methyl ester
