# Supplementary material for: Impact of Mutations in the NCAPG and MSTN Genes on Body Composition, Structural Properties of Skeletal Muscle, Its Fatty Acid Composition, and Meat Quality of Bulls from a Charolais × Holstein F2 Cross
Source: Int J Mol Sci. 2026 Jan 15;27(2):882. doi: 10.3390/ijms27020882 (PMC12841407; doi:10.3390/ijms27020882)
Supplement: Supplementary file 1 [file ijms-27-00882-s001.zip › ijms-4036322-supplementary.pdf]

Supplemental Tables

Impact of mutations in the *NCAPG* and *MSTN* gene on body composition, structural properties of skeletal muscle, its fatty acid composition and meat quality of bulls from a Charolais × Holstein F_2-cross

Elke Albrecht, Praveen Krishna Chitneedi, Dirk Dannenberger, Christa Kühn and Steffen Maak

Table S1. Descriptive statistics of carcass traits and allele substitution effects of MSTN Q204X and NCAPG I442M mutation in F_2-bulls (n = 241) of a Charolais × Holstein cross population.

	Descriptive statistics						Allele substitution effects					
							MSTN			*NCAPG*		
Trait	Mean	SD	Median	IQR	Min	Max	A	SE	*P*-value	A	SE	*P*-value
Birth weight, kg	47.3	6.4	47.5	8.0	31.0	71.0	2.26	0.82	0.0056	3.11	0.58	8.76E-08
Slaughter weight, kg	693.4	53.1	693.0	67.0	537.0	818.0	0.05	7.28	0.9950	24.56	5.03	1.04E-06
Dressing percentage, %	57.85	1.82	57.86	2.51	53.01	62.60	2.33	0.26	3.10E-19	-0.16	0.17	0.3489
Carcass length, cm	140.6	4.4	141.0	5.0	127.0	159.0	-0.67	0.61	0.2684	2.04	0.42	1.01E-06
Hot carcass weight (HCW), kg	401.2	33.2	400.9	42.0	305.4	489.0	16.05	4.58	4.55E-04	13.26	3.16	2.76E-05
Mesenteric fat, % of HCW	1.38	0.47	1.32	0.60	0.15	3.44	-0.11	0.07	0.0919	-0.14	0.04	0.0011
Omental fat, % of HCW	2.44	0.64	2.43	0.90	0.95	4.85	-0.20	0.09	0.0222	-0.30	0.06	2.57E-07
Perirenal fat, % of HCW	2.37	0.59	2.35	0.80	0.91	4.07	-0.35	0.08	1.06E-05	-0.29	0.06	2.13E-07
Cold carcass weight (CCW), kg	393.5	32.8	393.6	45.3	301.3	472.8	17.05	4.56	1.86E-04	13.52	3.16	1.91E-05
Meat, % of CCW	77.14	1.54	77.11	2.30	72.01	81.29	1.89	0.20	5.44E-21	-0.03	0.14	0.8314
SCF, % of CCW	5.70	1.48	5.57	1.92	2.19	11.74	-1.12	0.18	9.33E-10	-0.80	0.13	9.05E-10
Bones, % of CCW	14.69	1.27	14.56	1.70	11.03	19.05	-0.65	0.17	9.41E-05	0.75	0.12	2.73E-10
Tendons, % of CCW	2.46	0.29	2.42	0.42	1.68	3.28	-0.14	0.04	5.02E-04	0.08	0.03	0.0018
Carcass protein, %	14.52	0.81	14.60	1.10	11.94	16.40	0.88	0.11	7.92E-17	0.30	0.07	2.63E-05
Carcass fat, %	16.42	3.90	16.45	5.31	6.98	27.99	-2.91	0.47	9.07E-10	-1.98	0.33	3.07E-09
Daily gain of protein, g	100.0	12.5	99.4	16.0	69.5	127.6	10.86	1.53	1.42E-12	5.42	1.10	9.18E-07
Daily gain of fat, g	117.9	28.8	117.2	37.1	43.7	195.8	-16.67	3.70	6.73E-06	-9.90	2.43	4.62E-05

SD – standard deviation, IQR – interquartile range, Min – minimum, Max – maximum, A – allele substitution effect, SE – standard error

Table S2. Descriptive statistics of back and round composition and allele substitution effects of MSTN Q204X and NCAPG I442M mutation in F_2-bulls of a Charolais × Holstein cross population.

		Descriptive statistics						Allele substitution effects					
								MSTN			***NCAPG***		
Trait	n	Mean	SD	Median	IQR	Min	Max	A	SE	*P*-value	A	SE	*P*-value
Back (left carcass half)													
Weight, kg	241	16.10	1.70	16.13	2.26	9.68	20.66	0.77	0.24	0.0014	0.34	0.16	0.0332
Meat, kg	241	11.55	1.32	11.61	1.78	5.59	14.83	0.82	0.19	1.33E-05	0.32	0.12	0.0101
Subcutaneous fat, kg	241	1.35	0.48	1.32	0.67	0.23	2.60	-0.16	0.06	0.0120	-0.17	0.04	5.35E-05
Bones, kg	241	2.82	0.37	2.79	0.51	1.89	3.93	0.08	0.05	0.0845	0.17	0.04	1.04E-06
Tendons, kg	241	0.38	0.08	0.37	0.11	0.20	0.67	0.02	0.01	0.0870	0.01	0.01	0.3882
Water, %	241	60.83	4.54	60.97	5.94	49.80	72.34	2.94	0.55	8.22E-08	1.83	0.38	1.79E-06
Protein, %	241	17.96	1.34	18.18	1.59	12.45	20.88	0.96	0.17	3.56E-08	0.61	0.12	7.73E-07
Fat, %	241	20.40	5.61	19.92	6.92	6.89	34.92	-3.92	0.70	2.63E-08	-2.19	0.49	6.84E-06
Ash, %	241	0.83	0.07	0.84	0.08	0.64	1.00	0.04	0.01	1.95E-07	0.03	0.01	2.99E-06
Round (left carcass half)													
Weight, kg	241	52.45	4.87	52.30	6.50	40.10	65.90	3.61	0.65	2.94E-08	2.55	0.47	6.70E-08
Length, cm	238	75.91	2.76	76.00	4.00	70.00	91.00	0.01	0.36	0.9718	1.45	0.25	1.01E-08
Max. circumference, cm	238	127.2	4.8	127.00	6.00	114.0	144.0	3.56	0.65	4.06E-08	2.37	0.47	3.76E-07
Meat, kg	241	42.55	4.42	42.12	6.07	31.93	54.73	4.13	0.59	2.12E-12	2.21	0.43	2.16E-07
Subcutaneous fat, kg	241	2.45	0.77	2.35	1.04	0.75	5.08	-0.44	0.10	6.17E-06	-0.28	0.06	1.14E-05
Bones, kg	241	6.62	0.74	6.59	0.99	4.59	8.69	-0.08	0.09	0.3965	0.54	0.07	7.91E-14
Tendons, kg	241	0.84	0.19	0.82	0.26	0.42	1.53	-0.01	0.03	0.7795	0.06	0.02	0.0017
Water, %	241	67.05	3.29	67.35	4.54	56.51	73.15	1.82	0.36	4.37E-07	1.12	0.24	5.03E-06
Protein, %	241	19.05	0.87	19.14	1.08	16.11	21.48	0.72	0.11	1.90E-10	0.33	0.08	1.71E-05
Fat, %	241	12.90	3.70	12.65	4.93	6.10	25.22	-2.71	0.47	6.29E-09	-1.35	0.32	2.06E-05
Ash, %	241	0.91	0.05	0.92	0.05	0.67	1.03	0.03	0.01	1.82E-06	0.02	0.00	1.96E-04

n – number of animals, SD – standard deviation, IQR – interquartile range, Min – minimum, Max – maximum, A – allele substitution effect, SE – standard error

Table S3. Descriptive statistics of *M. longissimus* and *M. semitendinosus* characteristics and allele substitution effects of MSTN Q204X and NCAPG I442M mutation in F_2-bulls of a Charolais × Holstein cross population.

		**Descriptive statistics**						**Allele substitution effects**					
								MSTN			***NCAPG***		
Trait	n	Mean	SD	Median	IQR	Min	Max	A	SE	*P*-value	A	SE	*P*-value
M. longissimus													
Length, cm	241	100.6	4.2	101.0	5.0	90.0	110.0	-0.61	0.57	0.2832	1.63	0.37	9.88E-06
Circumference, cm	241	41.60	4.09	41.00	3.00	35.00	93.00	1.55	0.60	0.0092	0.77	0.38	0.0392
Weight, kg	240	8.09	0.91	8.12	1.26	5.14	10.23	0.90	0.13	7.48E-13	0.38	0.09	1.51E-05
Cross sectional area, cm^2	240	101.7	12.3	101.2	15.3	67.0	141.5	12.10	1.60	4.23E-14	2.36	1.09	0.0305
Intramuscular fat content, %	241	3.38	1.70	3.02	2.00	0.77	10.68	-1.32	0.22	2.79E-09	-0.59	0.15	1.04E-04
Lightness, L*	241	36.15	2.20	36.50	3.20	28.09	41.64	0.35	0.32	0.2614	0.34	0.20	0.0904
Forced water loss, %	241	29.96	5.24	29.88	7.30	17.37	44.32	-0.56	0.67	0.4035	0.23	0.42	0.5800
Shear force 24 h, kg	240	16.30	2.82	16.00	4.05	9.30	26.90	-0.17	0.40	0.6709	0.22	0.25	0.3875
Shear force 14 d, kg	241	10.63	2.07	10.40	2.60	6.20	17.59	0.66	0.28	0.0198	0.40	0.18	0.0249
pH value, 24 hours p.m.	237	5.50	0.09	5.51	0.11	5.23	5.76	-0.013	0.011	0.2369	-0.001	0.007	0.8640
M. semitendinosus													
Intramuscular fat content, %	241	1.84	0.74	1.73	1.05	0.34	4.15	-0.40	0.10	3.77E-05	-0.30	0.07	7.47E-06
Lightness, L*	241	39.90	2.01	39.88	2.53	35.21	45.21	1.18	0.55	0.0320	0.23	0.19	0.2201
Forced water loss, %	241	36.69	6.08	37.59	8.06	17.96	54.05	1.13	0.74	0.1273	-0.30	0.47	0.5230
Shear force 24 h, kg	240	21.37	2.82	21.80	3.95	11.49	26.90	0.78	0.40	0.0519	0.12	0.25	0.6490
Shear force 14 d, kg	241	14.48	2.23	14.40	5.0	9.00	23.32	0.75	0.28	0.0078	0.03	0.19	0.8813

n – number of animals, SD – standard deviation, IQR – interquartile range, Min – minimum, Max – maximum, A – allele substitution effect, SE – standard error

Table S4. Descriptive statistics of muscle fiber composition and intramuscular fat characteristics of *M. longissimus* and allele substitution effects of MSTN Q204X and NCAPG I442M mutation in F_2-bulls of a Charolais × Holstein cross population.

		**Descriptive statistics**						**Allele substitution effects**					
								MSTN			***NCAPG***		
Trait	n	Mean	SD	Median	IQR	Min	Max	A	SE	*P*-value	A	SE	*P*-value
Muscle fiber (MF) number/cm²	224	36478	6985	35789	8106	22994	68532	-1453	1024	0.1559	2237	677	9.46E-04
MF cross sectional area (CSA), µm²	224	2843.8	536.8	2800.0	640.0	1459.0	4349.0	125.1	79.1	0.1138	-152.5	51.6	0.0031
Apparent total MF number, x10⁶	223	3.68	0.77	3.65	1.07	1.93	6.22	0.29	0.11	0.0078	0.31	0.07	1.99E-05
Nuclei per MF	224	1.04	0.21	1.02	0.26	0.19	1.63	0.05	0.03	0.1012	-0.02	0.02	0.1889
MF type IIb/x CSA, µm²	224	3501.4	698.1	3398.0	842.5	1867.0	6036.0	302.4	104.8	0.0039	-95.5	66.7	0.1522
Area percentage of type IIb/x, %	224	56.56	9.45	57.24	13.91	22.54	77.43	9.62	1.26	2.28E-14	1.59	0.82	0.0537
MF type IIa CSA, µm²*	224	2475.7	591.2	2409.5	767.0	1247.0	4012.0	-167.6	83.4	0.0446	-208.7	57.5	2.85E-04
Area percentage of type IIa, %	224	27.42	8.34	26.74	10.86	9.34	63.93	-7.12	1.07	2.30E-11	-0.48	0.71	0.4961
MF type I CSA, µm²	224	2007.1	474.7	1912.5	687.0	913.0	3483.0	-109.9	67.6	0.1041	-206.5	46.1	7.38E-06
Area percentage of type I, %	224	16.02	5.63	14.77	5.72	6.03	47.85	-2.54	0.82	0.0020	-1.13	0.53	0.0333
Intramuscular fat cell diameter, µm	227	90.23	9.65	91.06	13.53	59.75	118.31	-4.50	1.32	6.43E-04	-3.72	0.92	4.80E-05
Number of marbling flecks	227	582.6	196.6	572.7	237.0	108.7	1159.7	-76.7	25.2	0.0024	-36.5	16.9	0.0305
Distance of marbling flecks, mm	221	1.91	0.32	1.86	0.30	1.33	3.85	0.19	0.04	2.50E-06	0.01	0.03	0.5776
M. longissimus CSA, cm²	227	102.4	13.2	101.5	17.6	69.5	144.4	13.0	1.8	4.80E-13	4.2	1.2	5.56E-04
Marbling area, %	227	5.65	2.68	5.28	3.53	0.90	15.96	-2.05	0.36	1.53E-08	-1.01	0.25	4.28E-05
Maximal marbling fleck area, mm²	227	144.2	115.9	106.8	137.3	6.0	583.1	-39.0	16.8	0.0204	-14.8	10.7	0.1668

n – number of animals, SD – standard deviation, IQR – interquartile range, Min – minimum, Max – maximum, A – allele substitution effect, SE – standard error

Table S5. Descriptive statistics of fatty acid composition in *M. longissimus* and allele substitution effects of *MSTN Q204X* and *NCAPG I442M* mutation in F_2-bulls (n = 241) of a Charolais × Holstein cross population

	Descriptive statistics						Allele substitution effects					
							MSTN			NCAPG		
FA, g / 100 g total FA	Mean	SD	Median	IQR	Min	Max	A	SE	*P*-value	A	SE	*P*-value
C12:0	0.092	0.064	0.079	0.040	0.035	0.585	0.006	0.009	0.5120	0.009	0.005	0.0847
C14:0	2.975	0.696	2.888	0.942	1.523	6.260	-0.239	0.093	0.0104	-0.198	0.059	8.45E-04
C14:1c9	0.596	0.288	0.567	0.370	0.000	1.549	-0.084	0.033	0.0116	-0.075	0.022	8.20E-04
C15:0	0.425	0.192	0.365	0.124	0.123	1.210	-0.002	0.022	0.9318	-0.007	0.014	0.6004
C15:1c10	0.097	0.097	0.076	0.141	0.000	0.689	0.033	0.012	0.0070	0.015	0.008	0.0441
C16:0	28.48	1.89	28.45	2.37	22.67	33.59	-1.16	0.26	8.95E-06	-0.37	0.16	0.0233
C16:1c9	3.811	0.766	3.807	1.150	1.866	5.611	-0.435	0.097	7.31E-06	-0.267	0.060	9.16E-06
C17:0	1.164	0.250	1.142	0.317	0.651	2.281	0.065	0.028	0.0200	-0.012	0.018	0.4965
C17:1c10	0.734	0.244	0.748	0.301	0.058	1.337	0.018	0.026	0.4805	0.000	0.016	0.9956
C18:0	13.38	1.52	13.22	1.68	9.82	18.92	0.35	0.21	0.1017	0.32	0.14	0.0199
C18:1t11	0.951	0.377	0.869	0.410	0.367	2.888	0.077	0.046	0.0917	-0.039	0.029	0.1725
C18:1c9	34.10	2.92	34.18	3.97	24.63	41.87	-2.06	0.38	8.53E-08	-0.97	0.26	1.76E-04
C18:1c11	1.676	0.413	1.621	0.519	0.781	2.666	0.045	0.044	0.3139	-0.014	0.028	0.6182
C18:2t9,t12	0.130	0.090	0.112	0.076	0.000	0.546	0.021	0.011	0.0514	-0.008	0.007	0.2348
C18:2n-6	5.450	2.263	5.011	2.416	1.967	16.880	1.864	0.291	1.58E-10	0.850	0.196	1.42E-05
C18:3n-3	0.509	0.150	0.486	0.191	0.228	1.272	0.102	0.019	4.93E-08	0.055	0.012	1.08E-05
C18:4n-3	0.060	0.110	0.009	0.031	0.000	0.487	-0.001	0.010	0.9069	-0.006	0.007	0.4014
C20:0	0.099	0.045	0.087	0.034	0.000	0.350	0.011	0.005	0.0167	0.007	0.003	0.0206
C20:1c11	0.138	0.031	0.133	0.041	0.073	0.297	-0.013	0.004	0.0028	-0.006	0.003	0.0373
C20:3n-6	0.393	0.214	0.341	0.228	0.072	1.334	0.166	0.027	1.10E-09	0.072	0.018	9.40E-05
C20:4n-6	1.645	0.952	1.419	0.993	0.197	5.537	0.608	0.125	1.04E-06	0.390	0.086	5.25E-06
C20:5n-3	0.106	0.064	0.096	0.063	0.000	0.346	0.035	0.009	4.91E-05	0.024	0.006	3.38E-05
C21:0	0.015	0.017	0.013	0.018	0.000	0.175	0.004	0.002	0.0686	0.002	0.001	0.1405
C22:4n-6	0.294	0.149	0.254	0.163	0.029	1.019	0.084	0.019	1.18E-05	0.065	0.013	8.45E-07

C22:5n-3	0.317	0.178	0.276	0.184	0.043	1.145	0.103	0.023	1.17E-05	0.076	0.016	2.29E-06
C22:6n-3	0.030	0.025	0.025	0.031	0.000	0.132	0.013	0.004	3.23E-04	0.005	0.002	0.0270
C24:0	0.026	0.024	0.024	0.029	0.000	0.136	0.009	0.003	5.63E-04	0.007	0.002	2.68E-05
CLAc9t11	0.228	0.089	0.246	0.095	0.000	0.485	0.001	0.008	0.8870	-0.006	0.005	0.2623
C10:0	0.269	0.228	0.220	0.295	0.000	1.424	0.108	0.026	3.67E-05	0.047	0.017	0.0052
C18:3n-6	0.039	0.024	0.035	0.032	0.000	0.152	0.011	0.003	6.74E-04	0.005	0.002	0.0258
C20:2n-6	0.080	0.032	0.074	0.040	0.000	0.189	0.022	0.004	1.90E-07	0.009	0.003	0.0014
C22:2n-6	0.023	0.032	0.000	0.048	0.000	0.130	0.002	0.003	0.3786	0.000	0.002	0.8355
SFA[1]	47.03	2.56	47.08	3.49	40.14	54.85	-0.84	0.35	0.0173	-0.17	0.22	0.4441
MUFA[2]	43.63	3.38	44.13	4.50	32.21	51.62	-2.24	0.43	1.53E-07	-1.38	0.30	3.17E-06
PUFA[3]	9.332	3.886	8.562	4.271	3.131	27.650	3.053	0.507	1.68E-09	1.540	0.344	7.48E-06
n-3 PUFA[4]	1.049	0.402	1.003	0.495	0.380	2.671	0.272	0.053	2.42E-07	0.162	0.036	6.21E-06
n-6 PUFA[5]	7.924	3.537	7.226	3.753	2.299	24.763	2.757	0.457	1.63E-09	1.391	0.310	7.37E-06
n-6/n-3	7.566	1.396	7.756	2.173	4.418	11.217	0.470	0.129	2.70E-04	0.112	0.080	0.1626
Sum FA/100 g	2.466	1.179	2.145	1.040	0.704	7.650	-0.772	0.162	1.83E-06	-0.404	0.112	3.11E-04

FA – fatty acid, Min – minimum, Max – maximum, SD – standard deviation, A – allele substitution effect, SE – standard error,

[1] SFA – saturated fatty acids = C10:0+C11:0+C12:0+C13:0+C14:0+C15:0+C16:0+C17:0+C18:0+C20:0+C21:0+C22:0+C23:0+C24:0.

[2]MUFA – monounsaturated fatty acids = C14:1+C16:1+C17:1+C18:1cis-9+ΣC18:1trans-9-11+C20:1.

[3] PUFA - polyunsaturated fatty acids = n-3 FA+n-6 FA.

[4] n-3 PUFA = C20:3n-3+C22:6n-3+C22:5n-3+C20:5n-3+C18:4n-3+C18:3n-3.

[5] n-6 PUFA = C22:2n-6+C20:2n-6+C18:3n-6+C22:4n-6+C20:3n-6+C18:2n-6+C20:4n-6.
